# Current position of 5HT_3_ antagonists and the additional value of NK_1_ antagonists; a new class of antiemetics

**DOI:** 10.1038/sj.bjc.6601033

**Published:** 2003-06-10

**Authors:** R de Wit

**Affiliations:** 1 Department of Medical Oncology, Erasmus University Medical Center and Rotterdam Cancer Institute, P.O. Box 5201, 3008 AE Rotterdam, The Netherlands

**Keywords:** Antiemetics, 5HT_3_ antagonists, NK_1_ antagonists, neurokinine receptor antagonists, substance *P*

## Abstract

The advent of the 5HT_3_ receptor antagonists (5HT_3_ antagonists) in the 1990 s and the combination with dexamethasone has resulted in acute emesis protection in 70% of patients receiving highly emetogenic chemotherapy. Despite complete protection in the acute phase, however, 40% of patients as yet have symptoms in the delayed phase. 5HT_3_ antagonists and dexamethasone are only modestly effective in this delayed phase. Moreover, the antiemetic protection over repeated cycles is not sustained. Neurokinine 1 receptor antagonists (NK_1_ antagonists) belong to a new class of antiemetic agents that specifically target the NK_1_ receptor, which is involved in both the acute and, particularly, the delayed phase of emesis. Clinical studies have demonstrated that the addition of NK_1_ antagonists to dual therapy with a 5HT_3_ antagonist plus dexamethasone improves the acute emesis protection by a further 10–15%. In the delayed phase, the proportion of patients remaining free of emesis increases by even 20–30%. Since the effectiveness of this triplet combination was found to be sustained over six cycles of chemotherapy, the chance for an individual patient to remain completely protected during both the acute and the delayed phase over six chemotherapy cycles is nearly doubled.

Nausea and vomiting are considered as two of the most distressing side effects of anticancer chemotherapy. Chemotherapy-induced emesis has a serious impact on a patient's quality of life and the possibility to complete the planned chemotherapy successfully. Cisplatin is most commonly associated with profound nausea and vomiting, which follows a distinct pattern of an acute phase (0–24 h after the start of chemotherapy) and a delayed phase that is mostly defined as occurring on days 2–5 after the chemotherapy. Since severe emesis occurs in virtually all patients who receive a cisplatin dose of ⩾50 mg m^−2^, cisplatin-induced nausea and vomiting has served as the model to test anti-emetic agents for highly emetogenic chemotherapy (Verweij *et al*, 1996; Gralla *et al*, 1999).

## CURRENT ROLE OF 5HT_3_ ANTAGONISTS PLUS DEXAMETHASONE; ACUTE PHASE

Before the advent of the 5HT_3_ antagonists, nausea and vomiting were ranked as the two most distressing side effects of systemic chemotherapy (Coates *et al*, 1983). The use of 5HT_3_ antagonists (ondansetron, granisetron and tropisetron) has provided complete acute emesis protection in 50-70% of patients receiving a first cycle of cisplatin-based chemotherapy (Gralla *et al*, 1999). The addition of dexamethasone to 5HT_3_ antagonists has improved the complete protection rate by a further 10–15%, resulting in a total complete acute emesis protection in 65–80% of patients (Gralla *et al*, 1999).

## CROSSOVER BETWEEN 5HT_3_ ANTAGONISTS IN ACUTE EMESIS FAILURE

In view of the similarity in chemical structure of the available 5HT_3_ antagonists it has been assumed for many years, while these agents all act at the same receptor, that failure to one agent would predict subsequent failure to all 5HT_3_ antagonists. This was recently refuted in a randomised double-blind study in 45 patients who had acute emesis protection failure on ondansetron plus dexamethasone following cisplatin- or cyclophosphamide-based chemotherapy (De Wit *et al*, 2001). Patients were randomised to receive in the next cycle either granisetron 3 mg plus dexamethasone, or to continue on ondansetron 8 mg plus dexamethasone. A significant benefit from crossingover to granisetron was found; of 19 eligible patients who crossed over to granisetron, nine patients obtained as yet complete protection, whereas this was observed in one of 21 eligible patients who continued on ondansetron, *P*=0.005. Several large, well-designed, randomised studies have demonstrated equivalent antiemetic efficacy for ondansetron and granisetron (Ruff *et al*, 1994; Navari *et al*, 1995, Stewart *et al*, 1995; Perez *et al*, 1998). Since there appears to be no therapeutical difference between ondansetron doses ranging from 8 to 32 mg (Ruff *et al*, 1994; Italian Group of Antiemetic Research, 1995; Gandara *et al*, 1998), these results may indicate that there is no complete cross-resistance between 5HT_3_ antagonists, and that patients who have acute emesis protection failure on one 5HT_3_ antagonist could be considered to crossover to another 5HT_3_ antagonist.

Of note, there is one hypothetical alternative explanation for the observation. Ondansetron is primarily metabolised by cytochrome 450 enzymes, including CYP 2D6, for which genetic polymorphisms have been identified (Fischer *et al*, 1994; Dixon *et al*, 1995; Davis *et al*, 2001; Wilkinson 2001). Such enzyme polymorphisms appear not to account for granisetron, which is primarily metabolised by CYP 3 A (Bloomer *et al*, 1994). Suboptimal levels of ondansetron due to extensive or ultrarapid CYP 2D6 metabolism in patients may not have a detectable impact in phase III trials in unselected patients. In the above clinical trial in patients selected for reasons of protection failure on ondansetron however, the successful crossover may be related to differences in 5HT_3_ antagonist metabolism in these patients.

## 5HT_3_ ANTAGONISTS PLUS DEXAMETHASONE: DELAYED PHASE

Despite the efficacy of 5HT_3_ antagonists in the initial 24 h period after the start of chemotherapy, the therapeutical role in the delayed phase is rather limited. Studies have shown that 5HT_3_ antagonists are more active than placebo against delayed nausea and emesis, but these agents are not more active or inferior to dexamethasone alone (Jones *et al*, 1991; Navari *et al*, 1995). Also, dual therapy with a 5HT_3_ antagonist plus dexamethasone did not improve the results obtained with dexamethasonea a alone (Italian Group of Antiemetic Research 1995; Goedhals *et al*, 1998; Latreille *et al*, 1998). As a consequence, many investigators consider dexamethasone a standard therapy in the delayed phase, whereas others feel that dual therapy with a 5HT_3_ antagonist for several days might theoretically provide some additional benefit, and does little harm.

With the advent of the 5HT_3_ antagonists, the spectrum of side effects associated with chemotherapy has changed from profound emesis in the acute phase to insidious prolonged and often debilitating symptoms in the delayed phase. Despite complete protection in the acute phase, 40% of patients suffer from delayed nausea and vomiting (Morrow *et al*, 1996). As these symptoms occur mostly in the outpatient setting, there may be considerable underestimation of delayed emesis by health care professionals. It was recently reported that doctors and nurses predicted the absence of delayed nausea in 76%, and no delayed vomiting in 91% of outpatients, receiving a first cycle of moderately emetogenic chemotherapy, while the actual patient diaries revealed that the absence of delayed symptoms was only 43, and 59%, respectively, resulting in a 30% difference between perception and reality (Grunberg *et al*, 2002).

In 1983, before the introduction of the 5HT_3_ antagonists, Coates *et al* (1983) reported on a survey in Australia that ranked the side effects perceived by patients who were receiving chemotherapy. This study identified vomiting and nausea to be the two most distressing side-effects. In 1995, the study was repeated in the Netherlands in a total of 197 patients, all of whom had received 5HT_3_ antagonists, and 75% of whom in addition had received dexamethasone (de Boer-Dennert *et al*, 1997). At a mean number of four chemotherapy cycles at filling out the questionnaire, the patients still ranked nausea and vomiting to be number one and three of the most distressing side effects. These studies show that nausea and vomiting have remained a significant burden to patients.

## 5HT_3_ ANTAGONISTS ANTIEMETIC EFFICACY IS NOT MAINTAINED OVER REPEATED CYCLES

It is well recognised that the chance to develop nausea and vomiting increases with each subsequent cycle of chemotherapy. Part of this can be explained by anticipatory mechanisms. With each additional cycle, a patient may have a protection failure on that occasion. A patient who has experienced post-treatment nausea and emesis more than once or twice is prone to develop anticipatory symptoms, and will suffer from emesis again in the next cycles (Morrow *et al*, 1997).

Several studies have demonstrated that indeed, and despite the use of 5HT_3_ antagonist plus dexamethasone, with each subsequent cycle of chemotherapy, emesis protection decreases (de Wit *et al*, 1996, 1998). In the largest study in 125 patients receiving six cycles of cisplatin-based chemotherapy, despite dual therapy with granisetron and dexamethasone both in the acute and the delayed period, the initial complete acute emesis protection decreased from 66 to 39%, and for delayed emesis, the initial complete protection of 52% decreased to 43, or 21% in the sixth cycle, depending on the type of statistical analysis. In this study, it was also found that unsuccessful delayed emesis protection adversely influenced acute emesis protection in subsequent cycles of chemotherapy (de Wit *et al*, 1998). If delayed emesis protection is not improved, most patients will develop emesis, and in due course both in the acute and in the delayed phase.

## NEW DEVELOPMENTS: NK_1_ ANTAGONISTS

Substance P is an 11-amino-acid neuropeptide of the tachykinin family of peptides. It is found in the gut and central nervous system and can produce vomiting when injected into ferrets (Watson *et al*, 1995). The biological actions of substance P are mediated through a specific neuroreceptor, neurokinin 1 (NK_1_). A number of nonpeptide compounds that selectively block the NK_1_ receptor have been identified, and a typical feature of the NK_1_ antagonists was that these abolished emesis from a wide range of profound emetic stimuli, including apomorphine, morphine, nicotine, copper sulphate, ipecacuanha, radiation, cyclophosphamide, cisplatin, and motion in the ferret and the dog (Watson *et al*, 1995). This wide spectrum of antiemetic activity is not shared by serotonin- and dopamine receptor antagonists, and suggests that substance P may exert a critical role in the emetic reflex pathway and presents an appropriate target for therapeutical intervention (Hesketh *et al*, 1999). The potential value of the NK_1_ antagonists in the treatment of chemotherapy-induced emesis was first recognised in ferret studies, indicating that both acute and delayed cisplatin-induced emesis were completely abolished (Rudd *et al*, 1996). Preliminary observations suggested that similar benefits might be obtained in patients (Kris *et al*, 1997). A number of clinical studies have now confirmed the effectiveness of NK_1_ antagonists both in the acute and, particularly, the delayed phase.

## NK_1_ RECEPTOR ANTAGONISTS: ACUTE AND DELAYED EMESIS

The effectiveness of the addition of the NK_1_ antagonist CJ-11,974 to standard therapy with granisetron plus dexamethasone was investigated in a randomised, placebo-controlled, phase II study in a total of 61 patients receiving a first cycle of cisplatin-based chemotherapy (Hesketh *et al*, 1999). Granisetron and dexamethasone were administered once before the cisplatin infusion, neither drug was continued on subsequent days. CJ-11,974 100 mg, or placebo, was administered orally, before and 12 h after cisplatin and then twice daily on days 2–5. The primary endpoint was the percentage of patients who developed delayed emesis. In patients on CJ-11,974, complete control of delayed emesis was obtained in 68 *vs* 37% in the patients who received placebo. There was also a numerical advantage for complete control of emesis on day 1 in favour of the CJ-11,974 containing group, 86 *vs* 67%, but this difference did not reach statistical significance with this limited number of patients.

Simultaneously with this trial report, a randomised phase II study of the NK_1_ antagonist L-754,030 (also known as MK-869) was published (Navari *et al*, 1999). This trial involved 159 patients. The standard treatment of granisetron plus dexamethasone in all patients was identical to that used in the above-mentioned study. The patients were randomised to receive L-745,030 400 mg orally before cisplatin and 300 mg on days 2–5, L-745,030 400 mg before cisplatin followed by placebo on days 2–5, or placebo both before and after cisplatin. In the acute-emesis phase, 93% of the patients in the L-754,030 groups and 67% of those receiving granisetron plus dexamethasone plus placebo had no vomiting (*P*<0.001). In the delayed-emesis phase, 82% of the patients receiving L-754,030 on all 5 days, 78% of those receiving L-754,030 only on day 1, and 33% of those receiving standard therapy had no vomiting (*P*<0.01 for the comparison of the active drug groups *vs* placebo). The median nausea score in the delayed-emesis phase was significantly in favour of the active drug for 5 days group. The numerical 4 percentage points further improvement obtained by continued dosing of L-754,030 on days 2–5 as compared to L-754,030 only on day 1 did not reach statistical significance, which may have been attributed by the modest sample size of the study.

More recently, the preceding proof of concept studies conducted with L-745,030, and its water-soluble intravenous prodrug L-758,298, were published (Cocquyt *et al*, 2001; Van Belle *et al*, 2002). The rationale for combining NK_1_ antagonists with 5HT_3_ antagonists is based upon the results of these phase II studies. In the first study, L-758,298 was directly compared with ondansetron in a randomised double-blind study in 53 patients receiving a first cycle of cisplatin (Cocquyt *et al*, 2001). The study evaluated the prevention of both acute and delayed emesis. In the acute period, the proportion of patients without emesis in the L-752,298 and ondansetron groups was 37 and 52%, respectively. In the delayed period, the proportion of patients without emesis in the L-758,298 and ondansetron treatment groups was 72 and 30%, respectively (*P*=0.005). Hence, the single dose of L-758,298 substantially suppressed delayed emesis. Although L-758,298 also appeared to reduce acute emesis post-cisplatin, there was a numerical advantage in acute protection for ondansetron. The study also provided solid data to support the hypothesis that the transition from the acute emesis phase to the delayed phase starts considerably earlier than 24 h after cisplatin administration (Horgan *et al*, 2001). It was found that the time course of acute vomiting following L-758,298 and ondansetron was quite distinct. The median time to first vomiting was 4.46 and 12.25 h in the L-758,298 and ondansetron groups, respectively. All acute failures in the L-758,298 group occurred in the first 8 h. These results indicate that later, but not ‘early’ acute vomiting is primarily substance P mediated and that, conversely, serotonin-mediated mechanisms may play a more important role during the “early” acute period. This finding provides a strong rationale to combine 5HT_3_ antagonists and NK_1_ antagonists to optimise acute emesis control.

Further data to support triple therapy with a 5HT_3_ antagonist, dexamethasone plus an NK_1_ antagonist were obtained in a randomised study involving 177 cisplatin-naive patients (Van Belle *et al*, 2002). Patients were randomized to one of three groups as follows: group I received L-758,298 intravenously plus dexamethasone before cisplatin on day 1, followed by L-745,030 orally on days 2–5; group II received L-758,298 plus dexamethasone on day 1, followed by placebo on days 2–5; and group III received ondansetron i.v. plus dexamethasone on day 1, followed by placebo on days 2–5. Additional (rescue) medication was available for emesis or nausea at any time. The primary efficacy parameters were the proportions of patients with no emesis and the proportion of patients without emesis or rescue therapy on day 1, and on days 2-5. Since use of rescue therapy is a clear measure of existing nausea that interferes with daily life, this composite end point of complete response appropriately evaluates both vomiting and nausea parameters. Dual therapy with ondansetron and dexamethasone (group III) was superior in controlling acute emesis (83% no emesis and no rescue *vs* 40% in groups I and II combined, *P*< 0.001), whereas the proportions of patients with no emesis and no use of rescue medication in the delayed phase were significantly better in the L-758,298 plus continued dosing (59%) and L-758,298 single dose (46%) groups as compared with the ondansetron plus dexamethasone group (38%), *P*<0.05 for group I *vs* group III. The numerical advantage for continued dosing with L-745,030 did not reach statistical significance with this limited sample size.

In conclusion, both studies lend support to the proposition that the underlying mechanisms of acute and delayed emesis are different, that 5HT_3_ antagonists are active in the acute phase, that NK_1_ antagonists are active in both the acute and, particularly, the delayed phase, and that the results warrant to test the potential of the combined use of a 5HT_3_ antagonist plus an NK_1_ antagonist. The numerical advantages reported by Navari and by Van Belle, which were both in favour of continued dosing with L-745,030, suggest that continued dosing may further enhance control of delayed emesis.

The first of the subsequent phase II b studies, which has been published as a full paper, investigated the triple regimen of granisetron, L-745,030 and dexamethasone *vs* granisetron and dexamethasone alone (Campos *et al*, 2001). In this trial, a total of 351 patients were randomised to four groups: group I received granisetron plus dexamethasone pre-cisplatin followed by placebo on days 2–5; group II received granisetron/dexamethasone plus L-745,030 orally pre-cisplatin, followed by L-745,030 orally on days 2 to 5; group III received dexamethasone pre-cisplatin and L-745,030 the evening before and pre-cisplatin, plus continued dosing with L-745,030; group IV received dexamethasone plus L-745,030 pre-cisplatin, plus continued dosing with L-745,030. Triple therapy with L-745,030 provided 23% additional complete emesis protection as compared with dual therapy with granisetron plus dexamethasone alone, 80 *vs* 57%, respectively, *P*<0.05. The importance for keeping the 5HT_3_ antagonist in the regimen to be used in the acute phase was again shown by the inferior results in the acute phase in groups III and IV (43 and 46%, respectively, *P*<0.05 as compared with group II). In the delayed phase, all three groups receiving L-745,030 had superior protection as compared with group I; the proportion of patients without emesis in groups I, II, III and IV was 29, 63, 51 and 57%, respectively, *P*<0.01 for groups II, III and IV *vs* group I.

The study demonstrated with sufficient statistical power that the triple combination of a 5HT_3_ antagonist and dexamethasone, plus an NK_1_ antagonist provided the best protection against acute emesis, and also confirmed and extended the previous findings that with the use of an NK_1_ antagonist, the control of delayed emesis is substantially enhanced.

### NK_1_ receptor antagonist efficacy over repeated cycles

In a similar phase II b study design, comparing triple therapy with dual therapy, the sustainment of antiemetic efficacy was investigated during six cycles of cisplatin-based chemotherapy (de Wit *et al*, 2002). All 202 patients received intravenous ondansetron and oral dexamethasone pre-cisplatin, and oral dexamethasone on days 2–5 of each cycle. The L-745,030 used in this study was the definitive formulation, which has obtained the generic name aprepitant (Merck Inc., West Point PA, USA). Patients were assigned in a blinded fashion to receive one of the following three regimens; group I, aprepitant 375 mg before cisplatin on day 1 and aprepitant 250 mg on days 2–5 (*N*=35); group II, aprepitant 125 mg before cisplatin on day 1 and aprepitant 80 mg on days 2-5 (*N*=81); group III placebo before cisplatin on days 2–5 (*N*=86). During the study, new pharmacokinetic data with the final formulation in healthy subjects became available that showed that the plasma levels of aprepitant attained with the 375 mg regimen were higher than expected, and a pharmacokinetic interaction was found to exist between the 375 mg regimen and dexamethasone. The 375/250 mg dose regimen (group I) was therefore discontinued. The primary end point was complete response (no emesis and no rescue therapy) during the overall period, defined as 0–120 h post-cisplatin (all of 5 days analysis). In view of the discontinuation of group I, the efficacy evaluations focused on the aprepitant 125/80 mg dose (group II) compared with standard therapy (group III). A cumulative probability analysis using the model for transitional probabilities was used to analyse the data, as was also applied in the previous multiple cycle studies with 5HT_3_ antagonists (de Wit *et al*, 1996, 1998). In the first cycle, the percentages of patients with complete response (0–120 h) were 64% in the aprepitant 125/80 mg (plus standard therapy) group and 49% in the standard therapy alone group, *P*<0.05. Thereafter, the percentages for aprepitant 125/80 mg ranged from 64% in cycle 2 to 59% by cycle 6, and those for standard therapy further declined to 34% by cycle 6. Statistical significance was achieved for between-treatment differences for cycles 5 and 6. Results for aprepitant 375/250 mg were similar to those obtained for aprepitant 125/80 mg. The reasons for discontinuation and the drop-out rates were comparable across the treatment groups.

Hence, the efficacy of the triple therapy was sustained through all six cycles, unlike that of the 5HT_3_ antagonist plus dexamethasone standard therapy, which further decreased. The benefit of triple therapy over standard therapy was clinically apparent as an almost two-fold increase (from 34 to 59%) in a patient's chance of remaining free of emesis and of the need for rescue medication during all of 5 days post-treatment and through six cycles of cisplatin-based chemotherapy.

## CONCLUSIONS

With the introduction of the 5HT_3_ antagonists in the 1990s and the combination with dexamethasone, complete acute emesis protection became possible in 70% of patients receiving a first cycle of highly emetogenic chemotherapy. 5HT_3_ antagonists and dexamethasone, however, are not very effective in the delayed phase. Even following complete protection in the acute phase, 40% of patients as yet have delayed symptoms, which interfere with daily life. As these symptoms typically occur in the outpatient setting, the impact is underestimated by health care professionals. Moreover, the incidence of both acute and delayed emesis increases with each cycle of chemotherapy, and after six cycles of emetogenic chemotherapy, the greater majority of patients suffer from delayed emesis and more than half of the patients have acute emesis as well, despite the use of dual therapy with a 5HT_3_ antagonist plus dexamethasone.

Recent data have shown that besides serotonin-mediated mechanisms in acute emesis (where 5HT_3_ antagonists fulfil their efficacy), substance P is another neuropeptide with a major role in the acute as well as, particularly, the delayed phase of emesis. Substance P exerts its effects by binding to a specific neuroreceptor, NK_1_. A number of nonpeptide compounds that selectively block the NK_1_ receptor have now been identified. Most of the data have been obtained with L-745,030, also known as MK-869, and the current generic name aprepitant. Studies in highly emetogenic chemotherapy have shown that the addition of aprepitant to dual therapy with a 5HT_3_ antagonist plus dexamethasone increases the acute emesis protection by a further 10–15%. The proportion of patients remaining free of emesis during the delayed emesis period, or when measured during all of 5 days following chemotherapy, increases by 20–30%. Moreover, the study results have shown that the protection against chemotherapy-induced nausea and vomiting is sustained over repeated cycles in patients receiving triple therapy including aprepitant. The confirmatory phase III trials have recently been completed and the data have been submitted for regulatory review, both in the US and in Europe.

The addition of these new NK_1_ antagonists to the existing 5HT_3_ plus dexamethasone combination appears to be another major leap forward in the successful protection against chemotherapy-induced emesis, which will become available in clinical practice soon.
